# Outpatient Treatment Based on Self-Management Strategies for Chronic Drooling in Two Children

**DOI:** 10.1007/s10882-017-9553-1

**Published:** 2017-05-31

**Authors:** Tessa W. P. de Bruijn, Jody Sohier, Jan J. W. van der Burg

**Affiliations:** 1 Department of Pediatric Rehabilitation, Sint Maartenskliniek, Postbus 9011, 6500 GM Nijmegen, The Netherlands; 2 Department of Pedagogical and Educational Sciences, Radboud University, Postbus 9104, 6500 HE Nijmegen, The Netherlands

**Keywords:** Drooling, Behavioural treatment, Self-management, Outpatient treatment

## Abstract

Drooling is a distressing condition, which is often caused by reduced oral motor control associated with a neurological disorder. It has significant medical, practical and psychosocial impact on children or youth and their families. Therefore, treatment is necessary. Although behavioural therapy for drooling shows promising results, it is generally time- and cost-intensive. For this reason, alternative ways to provide behavioural treatment for chronic drooling need to be explored. In a pair of case studies, the feasibility and potential of an outpatient variant of a behavioural treatment programme for drooling based on self-management strategies was researched with two children with oral motor difficulties. In a three week programme, these children were taught to perform a self-management routine in order to achieve saliva control during regular visits to the child rehabilitation centre. In addition, their parents and teachers were taught to prompt the self-management routine and instructed to provide additional practice at home and at school. In doing so, they were offered support by means of telehealth and personal contact. At the end of the treatment programme, both children showed a significant decrease in drooling severity. Their parents and teachers were satisfied with the treatment effect. Although the present treatment programme showed promising results, further adaptions are necessary to make the treatment programme more widely accessible.

Many children with developmental and physical disabilities, and occasionally also children with no obvious disability, do not develop adequate saliva control due to limited oral motor control and/or limited oral sensory awareness (Blasco and Allaire 1992; Burgmayer and Jung 1983; Talmon et al. 1981). Chronic drooling is a distressing condition as it can significantly affect health, participation, and social and emotional functioning. Treatment options for drooling vary from surgical and pharmacological interventions to the use of intra-oral devices, oral motor therapies, behavioural interventions, and proper body positioning. The effectiveness of these interventions has been reviewed in a number of studies (e.g., Walshe et al. 2012). There is a general consensus that treatment should be initially conservative, and if necessary, progress to more invasive procedures. For this reason, behavioural therapy for drooling is an option that should be considered before more invasive kinds of treatment (Blasco 2010; Brei 2003; Meningaud et al. 2006). For children with oral-motor problems and normal intelligence or mild intellectual disabilities, Van der Burg et al. (2007) put forward that behavioural therapies based on self-management procedures may be applicable. The aim of teaching the child self-management strategies is to develop internal control over the target behaviour (e.g., swallowing and wiping) instead of external social or automatic control (e.g., verbal prompts or auditory cues from a device). From this kind of behavioural therapy, the child learns to self-control and self-evaluate his/her physical appearance and saliva loss, and to prevent or initiate an appropriate response to drooling (Van der Burg et al. 2009). However scarce, previous studies using self-management techniques to treat drooling have yielded positive results (Dunn et al. 1987; Thorbecke and Jackson 1982; Van der Burg et al. 2009; 2016).

Van der Burg et al. (2009) developed a centre-based treatment programme for chronic drooling based on self-management techniques. In a three week inpatient intervention period at a child rehabilitation centre, participants were taught to perform self-management skills for saliva control and learned to remain dry for increasing time intervals. This programme was evaluated in a case series with 10 children (aged between 7;0 and 19;9 years) with neurodevelopmental disabilities. Following treatment, all participants were able to remain dry for 30–60 min while being engaged in daily activities. In addition, generalisation to the classroom at their own school occurred for all participants. Three participants were able to maintain self-management skills at 6 and 24 weeks follow-up. Seven participants failed to maintain these skills either 6 or 24 weeks after treatment. In order to improve effectiveness, generalisation, and maintenance, Van der Burg et al. (2016) elaborated the original inpatient treatment programme with differential (self-)reinforcement of swallowing, controlling, and wiping behaviour and explicit formulation of motivational factors by the participants. In addition, the parents and teachers were taught to provide instruction to their children and instructed to continue practising the self-management routine at home and at school after discharge. In another case series, this elaborated treatment programme was evaluated in 10 children (aged between 5;11 and 14;2) with oral-motor problems and normal intelligence or mild intellectual disabilities. The programme proved to be successful for 8 out of 10 participants. These participants were able to remain dry for a minimum of 30 min while being engaged in daily activities. Moreover, maintenance of the positive treatment effect 6 weeks after treatment was demonstrated by 6 participants and four of them maintained the treatment effect until 24 weeks follow-up.

Although this self-management programme for chronic drooling yielded promising results, these kind of inpatient interventions are generally time- and cost-intensive and therefore might not be cost-effective. Van der Burg et al. (2016) discussed that in many countries, such low tech, non-medical interventions are generally not conducted in an inpatient setting. An effective self-management treatment for drooling might, for this reason, not be available for some children suffering from chronic drooling. Consequently, there remains a need for an efficacious and cost-effective way to deliver self-management therapy for drooling.

For the present study, the self-management procedure of Van der Burg et al. (2016) was adapted with the aim of developing such a cost-effective, efficacious and feasible treatment programme for chronic drooling. The substitution of inpatient care to outpatient care is often seen as a means to reduce high costs (Vitikainen et al. 2010). Also, as the aim of the self-management programme is to prolong the ability of a child to remain dry during daily activities, it can be argued that provision of the intervention in the home and school situation is preferable above centre-based treatment. This allows for the realisation of teachable moments in day-to-day situations (Vismara et al. 2012). Therefore, the adapted treatment programme comprised outpatient self-management treatment provided by a behavioural therapist at a child rehabilitation centre for substantially less treatment time than in the original inpatient programme. Parallel to and following the outpatient centre-based treatment, additional training was provided by parents at home. In addition, following the outpatient treatment, continued practice was provided by the teacher at school. A distinctive quality of such a combination of an outpatient and home- and school-based treatment programme is that the participants will directly start applying the newly acquired skills in their natural environment and parents and teachers may become natural providers of reinforcement. As training takes place in a variety of settings, including the child’s natural environment, problems in generalisation and maintenance may be circumvented (Stokes and Osnes 1989).

The effect of a home- and school-based treatment depends to a great extent on the commitment of parents and teachers to provide training in the home and school situation. The parents and teachers, likely, will have become used to drooling throughout the years. Consequently, they might be less perceptive to the condition and not provide adequate, timely and sufficient responses to drooling. To focus attention on specific situations in which drooling is a problem and maximise parents’ cooperation, it seems important that they are actively engaged in the goal setting and evaluation of the treatment programme. In addition, to ensure maximal compliance of parents and teachers to providing treatment and procedural integrity, detailed instruction and coaching of parents seems crucial. To support parents and teachers in implementing a behavioural procedure at home or at school, a telehealth provision, in addition to personal contact and contact via telephone and email, may be helpful. Telehealth allows for easy access to the behavioural therapist for advice, consultation and ongoing support over a geographical distance (Vismara et al. 2012). However, it should be considered that, as Salmone and Maurizio Arduino (2017) put forward, not every parent may be prepared to be enrolled in support via telehealth.

In conclusion, the goal of the present study was to explore if the elements of the original treatment programme of Van der Burg et al. (2016) were applicable in an outpatient variant with continued practice at home and at school. Parents and teachers were supported in providing practice by ongoing coaching, also offered through a telehealth provision. In a pair of case studies, it was researched if the outpatient variant of the original treatment procedure was feasible and demonstrated promising effects in decreasing drooling severity in two children, and if effects were maintained. In addition, it was evaluated if parents and teachers were willing and able to follow through with the procedures at home and at school. Furthermore, the satisfaction of the parents’ and teachers’ with the treatment effect was evaluated.

## Method

### Participants

Between October 2015 and July 2016, two children participated in the present study. Inclusion criteria were: (a) severe drooling, defined as a score of 3 or higher on the Teacher Drool Scale (TDS; Camp-Bruno et al. 1989), indicating at least ‘occasional drooling, intermittent all day’, (b) a developmental age of 6 years or higher, (c) the ability to close their mouth and swallow on demand, (d) the ability to check and clean their mouth/chin or to ask or prompt the trainer to wipe their face dry, (e) the ability to maintain an upright seated position in a (adapted) chair, (f) no uncontrolled (epileptic) seizures, (g) no severe aggressive or hyperactive behaviour, (h) no medical treatment for drooling in the 6 months preceding participation in the present study, (i) some overt awareness (i.e., comments of the participant) of practical and social (adverse) consequences of drooling, (j) intrinsic motivation to achieve saliva control, and, finally, (k) parents and teachers had to be willing and have the possibility to train with the child on a daily basis.

The first participant, A, was a boy aged 7;8 years. He had mild oral motor problems, which consisted of severe malocclusion and low muscle tone in the mouth and cheeks. He had no motor or intellectual disability. He was included in this study in October 2015. Pre-baseline drooling severity was scored 4 on the TDS, indicating frequent drooling, but not profuse.

The second participant, B, was a girl aged 9;10 years. B was diagnosed with spastic diplegia cerebral palsy. Her motor functioning was classified as level II on the Gross Motor Function Classification System (GMFCS; Palisano et al. 1997) indicating that she was able to walk in most settings and climb stairs holding onto a railing, but experienced difficulty walking long distances and balancing on uneven terrain, inclines, in crowded areas, or confined spaces. She had minimal ability to perform gross motor skills such as running and jumping (Cerebral Palsy Alliance 2016a). B had moderate oral motor problems, consisting of limited strength in her lips and tongue, malocclusion, and insufficient automated swallowing. B communicated through speech. Her everyday communication was classified as level I on the Communication Function Classification System (CFCS; Hidecker et al. 2011), indicating that she independently and effectively alternated between being a sender and receiver of information with most people in most environments (Cerebral Palsy Alliance 2016b). B had a learning disability, but no intellectual disability. B was previously treated (more than six months prior to the behavioural treatment) for drooling with Botulinum toxin injections in the submandibular glands. She was included in the present study in February 2016. Pre-baseline drooling severity was scored 3–4 on the TDS, indicating occasional to frequent drooling, but not profuse. Both A and B attended a Dutch mainstream primary school.

Prior to the start of the treatment, informed written consent was obtained from the parents of both participants included in the study.

### Setting and Materials

The treatment was initially provided at the Child Rehabilitation Department of Sint Maartenskliniek (Nijmegen, the Netherlands). Treatment sessions and data collection took place in a child-friendly decorated room with a table and three chairs. Parents and children were asked to bring schoolwork and play materials to the rehabilitation centre as to enable the trainer to provide training and collect data during a variety of activities, which were similar to the daily activities of the participant and, therefore, could facilitate generalisation (Stokes and Osnes 1989). A laptop with DVD-player and internet connection were present in the room and there was access to a Nintendo Wii game console and a ping-pong table outside of the room. A video camera with a tripod, a stopwatch and a notebook were available for the purpose of data collection. A description of the training procedure, a (hand) mirror, tissues, a clock or a watch, some pencils, and stickers were present in all training situations. Training sessions at home and at school were provided during a variety of activities in a variety of settings. A so-called ‘swallowing-report’ was used for registration purposes and taken to and from the rehabilitation centre and home or school with the participant. The parents and teachers of both participants had access to a video camera or smartphone with camera function and a computer with internet connection.

### Dependent Variables

Drooling was defined as saliva, either a drop or a string, which is present beneath the lower lip line or falling directly from the mouth and which is not acted upon by the participant (i.e., by cleaning his/her face or clothes) within a period of two seconds. The dependent variable was latency in minutes of being dry (i.e., non-drooling) while performing daily activities. Latency measurements were scheduled throughout all study phases and were all video-taped. The activities during the latency recording sessions were at random selected from the participant’s daily school and leisure activities. At the start of each latency recording session the trainer said: “When you are dry, we can start”. After the participant checked and, if necessary, wiped his/her chin, the latency recording was started. As soon as drooling was observed, the latency recording was stopped. During the latency recordings neither instructions nor comments on drooling were provided to the participants.

### Design and Data-Collection

For both cases, data were collected during four study phases: baseline, intervention, post-intervention, and follow-up with data-collection 6 and 24 weeks after the centre-based treatment. There was a partial overlap in these phases across the participants: the follow-up at 24 weeks for A overlapped in time with the baseline phase for B. The latency of being dry was measured repeatedly during each study phase.

#### Baseline

To determine the level of pre-treatment drooling, baseline latency recordings were carried out at the rehabilitation centre and scheduled on two consecutive days before the start of the treatment. Baseline recordings lasted a maximum of 15 min, so that latency scores could be gathered over different kinds of activities over the course of two days. The number of baseline recordings varied per participant: A had 11 recordings, B had 12 recordings. The self-management intervention was started after visual analysis showed a stable trend in latency scores.

#### Self-Management Intervention

Two one-to-one sessions of 90 min were scheduled daily in the morning. Each session consisted of one or more training trials, depending on the time interval that was set. In addition, one latency recording was scheduled daily, at the end of the second training session. The duration of the latency recording was equal to the interval length which was trained at the time. Once the participant repeatedly had remained dry during a 30-min training trial, latency recordings with a maximum of 45 min were scheduled. This maximum was chosen to assure a minimum of two training trials and one latency recording per day. To prevent latency scores to be influenced by the kind of activity that was performed during latency recordings, activities during latency recordings were at random selected from the activities performed during baseline recordings.

#### Post-Intervention and Follow-Up

In the week following the last training session at the rehabilitation centre, latency was assessed at school during four recordings of 45 min, which were scheduled on one day. These recordings were repeated during the 6 and 24 weeks follow-ups.

### Reliability

A second rater independently, but not simultaneously, scored a third of all (video-taped) latency recordings, equally distributed across the participants and the study phases, in a random order. The interrater agreement was assessed using a two-way random, agreement, single-measures intraclass correlation coefficient (ICC; McGraw and Wong 1996). The overall ICC was in the excellent range, ICC = 0.97, indicating that the raters had almost perfect agreement on latency scores for the participants (Cicchetti 1994). The ICC’s for each study phase were in the excellent to perfect agreement range, respectively ICC = 0.98 (baseline), ICC = 1.00 (intervention), ICC = 0.85 (post-intervention and follow-up combined). In addition, the ICC was calculated for the latency recordings of both A and B. The resulting ICC for the latency recordings of A indicated perfect agreement between raters, ICC = 1.00. The ICC for the latency recordings of B was also high, ICC = 0.93, indicating excellent agreement.

### Procedure

#### Self-Management Training

The training of the self-management procedure at the child rehabilitation centre covered three weeks. During the first two weeks of the centre-based treatment, the participants and one of the parents visited the child rehabilitation centre every morning on weekdays. During the third week of the intervention, the participants visited the centre two or three mornings, depending on their progress. As the participants had to travel for about one hour to and from the rehabilitation centre, they did not go to school on treatment days. At the rehabilitation centre, two one-to-one sessions of 90 min were scheduled every morning. During latency recordings and training sessions the participants performed daily activities, such as schoolwork and leisure activities. Gross motor (e.g., playing outside and playing Wii-games) and fine motor activities (e.g., school work and colouring) were alternated.

After the baseline latency recordings and before the start of the intervention, the participant’s personal motivational factors for being dry, such as “my drawings will not get wet”, were formulated and written down on a ‘motivation-list’. The participants were also encouraged to ask their parents, friends, and other family members why they would like them to be dry and add these reasons to the list. The aim of the motivation-list was to get the participants intrinsically motivated to obtain saliva control. The motivation-list was added to the swallowing-report. During the intervention, additional comments were occasionally added to the motivation-list. The centre-based self-management intervention consisted of three training phases, with each phase roughly covering one week.

##### Phase 1

During the first training phase, the participants were taught to perform the self-management routine, which consisted of swallowing, checking if their chin was dry, and wiping if their chin was wet. This self-management routine was practised daily during multiple trials with increasing time intervals. Time intervals were set before intervention and were respectively ½, 1, 2, 5, 10, 15, 20, 25 and 30 min. The first training trial started with a time interval just below the mean latency of being dry as established during the baseline phase, with a maximum of 5 min. For A, initial training trials were set at 5 min. For B, initial trials were set at 2 min intervals. When the participants successfully remained dry during three consecutive trials, the next longer time interval was set for the subsequent trial. When a participant failed to remain dry in three consecutive trials or three times during five trials, the previous time interval was set again. Note that the support from the trainer gradually decreased: the longer the time intervals that were practised, the lower the frequency of explicit instruction that was provided to the participants. For a detailed description of a treatment session see Van der Burg et al. (2016; for a flowchart of the procedure of phase 1, see Appendix).

##### Phase 2

Once the self-management skills were familiarised and the participants were able to remain dry for 15 min intervals, the second phase of the intervention would come in effect. For A, this was at the fourth day of intervention; for B, this was at the start of the second week of the intervention. In the first session of each day, the procedure of phase 1 was continued. During the second session, self-instruction according to a script of pictographs depicting the self-management procedure and differential evaluation and reinforcement of the performance of the self-management skills was practised during 10–15 min intervals. Initially, the trainer guided the participant through this self-evaluation and self-registration. During subsequent trials, the participant learned to perform this procedure without help of the trainer. However, to prevent the participant to over- or underestimate his or her performance, the opinion of trainer remained decisive.

##### Phase 3

The third training phase covered the third week of the intervention. During this week, the participants would go to school again for two (B) to three (A) days and visit the rehabilitation centre on the other days. The trainer monitored the independent and adequate performance of the self-management skills and supported the participant by giving feedback and, if necessary, additional instructions. During training sessions, the time interval was no longer extended, but the self-management routine was automated by continuous repetition. The participants were eventually taught to whisper the self-instruction or use internal speech, so the use of self-instruction would not be inconvenient in the natural environment. They were instructed to use the self-management routine at school in order to stay dry. On Friday the trainer evaluated with the participants the use of the self-management routine at home and at school during the previous days.

#### Parent and Teacher Instruction and Coaching

After baseline, parents were instructed to occasionally provide positive feedback to the child during the day for being dry, swallowing or performing self-care (i.e., checking their chin and wiping). During the first phase of intervention, parents received verbal and written information about the behavioural principles that underlie the treatment procedure (i.e., instruction, feedback, and positive reinforcement). The last day of each week, parents were invited to join a training session. The trainer modelled the self-management procedure and guided the parents during several trials in practising the self-management procedure with their child. Verbal feedback was provided by the trainer on how parents instructed, provided feedback, and reinforced their child. During the present study, the digital healthcare platform Quli (https://www.quli.nl/) was used. Quli is designed to enable people to take control of their own care, by providing clients access to information and support in the home situation (Amarant Groep 2015). Quli was introduced to parents during the first phase of treatment; parents were provided instructions on how Quli could be used and they were familiarised with the use of Quli. The parents (and teachers) of both participants had a personal account on Quli, which they could (optionally) use to find information regarding the treatment and ask questions.

As the centre-based training was interrupted during the weekends, parents were instructed to initiate training sessions at home. After the first week of intervention, parents were instructed to initiate a minimum of three training trials per day with a time interval just below the longest time interval that was practiced at the centre. If the participant succeeded to remain dry, the parents could give him/her a sticker in his/her swallowing-report. If the participant did not succeed, the parents were instructed to mark a red cross, and provide brief negative verbal feedback. A written description of the treatment procedure and a video recording of a training session at the centre, in which the treatment procedure was shown, were available for the parents on Quli. In addition, parents had the possibility to stay in contact with the trainer through the message-function of Quli, in case they had any questions. Parents were asked to videotape one training trial at home during the weekends. After the weekend, the trainer evaluated this videotaped trial together with the parents and provided feedback. Furthermore, at the end of the first week of intervention, the participant’s teacher was telephoned and notified about the content of the first phase of the intervention and the progress of the participant.

During the second week of intervention, parents were instructed to provide one additional training session in the home situation in the afternoon. At the end of the second week, parents learned to implement the procedure for differential evaluation and reinforcement of the self-management skills and the use of the script of pictographs. They were instructed to practise the differential evaluation and reinforcement and the use of the script with their child, during two 10 min intervals a day. Additionally, they were asked to practise one trial a day in which the child was instructed to remain dry during an interval that equalled the interval that was practised at the centre. Parents were asked to videotape one trial in which they provided the differential evaluation and reinforcement of the self-management skills. After the weekend, this videotaped trial was again evaluated with the trainer and feedback was provided.

At the end of the second week of intervention, the teacher was also telephoned again. He/she was notified about the content of the training procedure during the second week of intervention, the progress of the child, and was provided with additional instructions. In addition, the teacher was verbally instructed (in much the same way as parents) to practise with the participant at school. The teacher obtained written instructions, just like the parents, and was invited to watch a videotaped training session through Quli.

At the end of phase 3, parents were instructed to continue to practise the self-management routine with their child at home during the first 6 weeks after the centre-based treatment by implementing differential reinforcement twice a day and one trial in which the participant was asked to remain dry for the maximum time interval achieved at the centre. They were instructed to initiate training sessions once a day on schooldays and three times a day in the weekends. In addition, they received tips and tricks on how to maintain motivation of the participant and treatment results. The teacher was instructed to practise the self-management procedure with the participant two times a day at school for the first 6 weeks after initial treatment. The swallowing-report was daily taken to school and back home by the participant for registration purposes. Parents and teachers were instructed to keep providing occasional positive feedback contingent on non-drooling and the performance of the self-management skills by the participant.

Three and 12 weeks after the end of the centre-based intervention, the trainer contacted the parents and teachers by telephone. In addition, the trainer visited the participant’s school after 6 and 24 weeks. Each contact, the progress of the participant and the current status of drooling was discussed with the parents and the teachers. If the positive effect of the treatment was maintained, the parents and teacher were instructed to gradually fade the frequency of practice at school or at home. If relapse occurred, the parents and teacher were advised to increase the frequency of practice at home and at school. In addition, during the school visits, the trainer observed the teacher practising with the participant and provided feedback. If necessary, additional instructions or tips and tricks were provided.

### Social Validity

In addition to the objective evaluation of the treatment effect through latency recordings, parents and teachers completed a Visual Analogue Scale (VAS) for drooling severity at home and at school for 10 to 14 consecutive days before baseline, and at post-intervention and follow-up at 6 and 24 weeks. The VAS is a 10 cm line on which drooling severity could be indicated with a score between 0 (no drooling) and 100 (very severe drooling). For each study phase the mean and standard deviation of the VAS scores were calculated. In accordance with Scheffer et al. (2010), a reduction of 2 standard deviations from the mean VAS score at baseline was considered a clinically significant change.

To maximise parents’ cooperation, they were included in the goal setting and evaluation of the treatment programme. For this purpose, an additional outcome measure, based on the assessment part of the Canadian Occupational Performance Measure (COPM; Law et al. 2005) was developed. Parents were asked to identify five daily activities of importance to the participant in which the saliva control was most problematic and/or drooling was especially undesirable. Similar to the COPM, their perception of the current performance of saliva control of their child during these activities and their personal satisfaction with this performance were rated on a 10-point scale, with 1 indicating *poor performance and low satisfaction*, respectively, and 10 indicating *good performance and high satisfaction*. Initial assessment took place before the start of the treatment (baseline). Reassessment took place directly after intervention (post-intervention phase) and at 6 and 24 weeks follow-up. For each participant, average performance and satisfaction scores over the five selected activities were calculated for each study phase. These average performance and satisfaction scores were used to calculate the performance and satisfaction change scores at post-intervention and follow-up compared to baseline (COPM 2016). As the procedure of calculating average performance and satisfaction scores was similar to the procedure of the COPM, a change score of two or more points between initial and outcome measurements was viewed as clinical relevant (Law et al. 2005). In the following, this outcome measure will be called the Measure of Performance and Satisfaction for Saliva Control (MPS-_saliva control_).

## Results

During the outpatient treatment programme, the feasibility was constantly evaluated using anecdotal information from the children and their parents and teachers. Both A and B mentioned that they liked coming to the rehabilitation centre, as they enjoyed the activities provided. Both children were motivated to practice to remain dry; they were seemingly proud that they were able to remain dry for increasing time intervals while performing the activities. A reported that prior to the treatment, he was avoided by other children because of his drooling. After the initial centre-based treatment, A mentioned that he was not avoided by other children anymore. Furthermore, he made positive remarks about his physical appearance and he liked it that his clothes did not get wet anymore. B enjoyed that her drawings and other crafts did no longer get wet. The centre-based training sessions were quite intensive for both children. They were not able to go to school during the three weeks of centre-based treatment as they were too tired. However, as the children completed schoolwork during training sessions, this did not cause them to fall behind on any schoolwork. Although both participants, especially A, did not like to be videotaped while performing activities, they were willing to follow through with the treatment, as both of them were motivated to achieve saliva control.

Both A and B were mostly accompanied by their mothers to the rehabilitation centre during the phase of centre-based intervention. The parents of both A and B reported to be surprised by the positive changes in saliva control directly after the start of the centre-based treatment. The mothers of A and B did find it quite intensive to travel to and from the rehabilitation centre on a daily basis, especially as they had other daily demands. For this reason, they reported that the centre-based treatment time should not have been longer than three weeks. In spite of the intensiveness of the centre-based treatment, parents of both participants were willing to follow through with the treatment because they experienced such positive changes in the saliva control of their child.

For both A and B, the home-based training sessions were also mostly supported by their mothers. Both mothers contacted the trainer by mail or telephone if they had any questions. Although Quli was offered as a means to find information regarding the treatment, both mothers preferred contact over the phone if they just wanted to ask a question. Both mothers reported that they found it sometimes hard to realise the desired amount of training sessions a day because of daily demands.

The teachers of both participants were positively engaged in the implementation of the self-management procedure at school. His teacher reported that A self-initiated the school-based training sessions. These training sessions were easily incorporated in his school programme. For B, training sessions on schooldays were scheduled throughout the day. Both teachers were willing and able to invest some time in the school-based training sessions at school as they found it important that the children achieved saliva control.

Three weeks after the intervention, A seemed to get annoyed with the explicit training trials at home and at school. As drooling only occurred sporadically at that time, scheduled practice at home and at school was gradually faded and eventually stopped around the 6 weeks follow-up sessions. B also got annoyed with the continuous training of the self-management procedure, three weeks after the outpatient treatment. As B had mastered the routine, it was proposed to decrease the amount of scheduled training sessions at home. In any case the parents and teachers were instructed to reinforce good performance of the self-management skills during the day and provide positive and if necessary some negative feedback accordingly. B got ill around the time of the 6 weeks follow-up. Consequently, a relapse in drooling severity was observed. For this reason, her parents and teacher were instructed to renew the scheduled training trials at home and at school. During the 24 weeks follow-up, B’s mother reported that renewed practice helped B to apply the self-management routine and remain dry again. As saliva control still was rather difficult for B at some times, her parents and teacher were instructed to continue practice at home and at school occasionally.

**Fig. 1:**
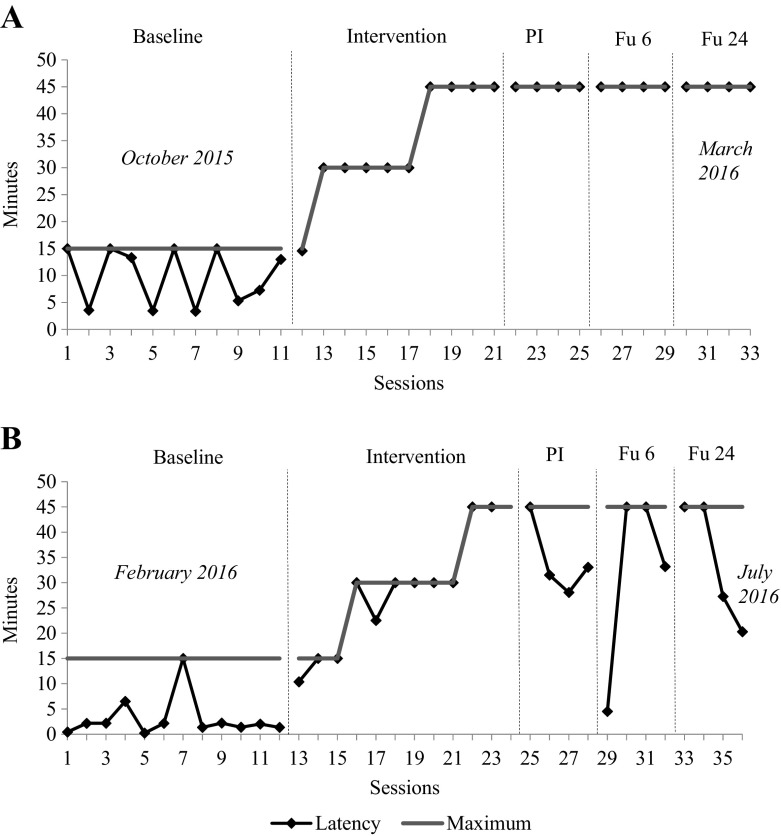
Individual latency scores in minutes of being dry during baseline, intervention, post-intervention (PI), and follow-up after 6 (Fu 6) and 24 (Fu 24) weeks. The solid vertical lines indicate the maximum interval lengths

The latency scores (i.e., the number of minutes of being dry) for each participant during baseline, intervention, post-intervention, and follow-up at 6 and 24 weeks are depicted in Fig. 1. Visual analysis revealed that latency scores for A at baseline were somewhat variable, with a slight upward trend at the end of the baseline phase. Four of the baseline measurements reached maximum latency of 15 min. Right after the start of the intervention phase, A was constantly able to remain dry for 15 min. In the next sessions, his latency scores showed a strong increase. From the 18th session on, his latency scores were also at least 45 min, which was the maximum time interval for latency recordings in the study. Right after intervention, A succeeded to remain dry for 45 min during four post-intervention trials at school. This was maintained at 6 and 24 weeks follow-up: latency scores of these phases were equivalent to the latency scores at the end of the intervention.

Baseline measures of B showed that at the start of the treatment, B was able to remain dry about 1 to 2 min. Latency scores during baseline showed some variability. During baseline measurements, B reached maximum latency only once. After the start of the intervention B was able to remain dry for 15 min. Her learning curve showed one small downturn at session 17, but showed a steady increase in latency scores throughout the intervention phase. After the three week intervention period, B was able to remain dry for 45 min while being engaged in daily activities. Latency scores at post-intervention and follow-up at 6 and 24 weeks suggested there was a partial loss of the treatment effect compared to the end of the intervention period. Even though latency scores during these study phases showed considerable variability, the level of saliva control remained well above baseline level.

Individual average VAS scores per study phase are provided in Table 1. Unfortunately, there were many missing values due to practical issues (i.e., days off from school etc.). Nevertheless, the change in VAS scores over the study phases seemed to be roughly congruent with the observed changes in latency scores. The parents’ and teacher’s reports on drooling severity of A showed a clinically significant decrease in drooling severity compared to baseline. Mean VAS scores at post-intervention and the follow-up 6 and 24 weeks after intervention, indicated drooling to be absent. For B, reports on drooling severity showed a continuing decrease over the subsequent study phases compared to baseline, however not clinically significant. The mean VAS scores for B have large standard deviations, indicating there was considerable variability in drooling severity over days. Generally, parents’ reports were comparable to the teacher’s reports. For B however, the VAS scores reported by parents at the 6 weeks follow-up showed a clinically significant increase in drooling severity compared to the post-intervention phase, although still below baseline level. The VAS scores reported by her teacher, on the contrary, remained stable.

**Table 1:** Mean and standard deviation of the VAS scores for baseline, intervention, post-intervention and follow-up at 6 (Fu 6) and 24 (Fu 24) weeks

		Baseline			Post-intervention			Fu 6			Fu 24		
Participant	Respondent	*n*	Mean (*SD*)		*n*	Mean (*SD*)		*n*	Mean (*SD*)		*n*	Mean (*SD*)	
A	Parents	7	55.57	(21.10)	14	1.07	(2.09)	14	0.21	(0.77)	14	0.00	(0.00)
	Teacher	4	40.50	(15.66)	4	0.00	(0.00)	8	0.00	(0.00)	8	0.00	(0.00)
B	Parents	14	56.64	(23.60)	14	15.29	(11.98)	14	52.36	(7.24)	14	38.50	(13.15)
	Teacher	6	57.33	(22.02)	8	27.38	(28.03)	9	24.11	(14.56)	5	36.80	(24.93)

The individual average performance and satisfaction scores on the MPS-_saliva control_ are presented in Table 2. For A, parents reported drooling to be most problematic or bothersome whilst playing football, watching television, writing, carting and reading. Prior to treatment, the average performance score for saliva control during these activities was 6.6. The corresponding average satisfaction score was 6.8. At the final reassessment the performance and satisfaction change scores were +3.4 and +3.2 respectively. These change scores indicate a clinically important improvement in the parental perception of the performance and their satisfaction with his saliva control. Most noteworthy, his parents reported maximum satisfaction and performance scores.

**Table 2:** Individual mean performance (P) and satisfaction (S) scores of saliva control reported on the MPS-_saliva control_ at baseline, post-intervention and follow-up at 6 (Fu 6) and 24 (Fu 24) weeks

	Baseline		Post-intervention		Fu 6		Fu 24	
Participant	P	S	P	S	P	S	P	S
A	6.6	6.8	10	10	10	10	10	10
B	2.8	3.6	8.6	8.6	7.6	7.6	8.2	8.0

With regard to B, parents mentioned drooling to be most problematic or bothersome whilst watching movies on the laptop, walking the dogs, watching television, talking enthusiastically and handing out treats to classmates or visitors at home. The initial average performance and satisfaction scores were respectively 2.8 and 3.6. Right after the three week intervention period (at post-intervention), both the average reported performance and satisfaction scores were 8.6. Parents reported a positive increase in performance and satisfaction regarding the saliva control of B on all five activities. The reported performance and satisfaction scores at the 6 week follow-up showed some relapse. However, during follow-up at 24 weeks, parents reported an average performance score of 8.2 and an average satisfaction score of 8.0. The performance and satisfaction change scores were respectively +5.4 and +4.4, illustrating clinically important improvement in performance and satisfaction.

## Discussion

In a pair of case studies, the outpatient variant of a self-management programme was provided to two children suffering from chronic drooling. Evaluation of the feasibility of the treatment programme put forward that the children, parents and teachers were all motivated and able to follow through with the present treatment programme. The daily travels to and from the rehabilitation clinic were found to be quite intensive. In addition, the parents of both A and B found it sometimes hard to realise the desired amount of training sessions at home. Nevertheless, the children, parents and teachers were motivated for the treatment because of the meaningful decreases in saliva loss, which were already visible directly after the start of the treatment.

Visual analyses of the latency scores indicate that after the implementation of the treatment programme, drooling severity was significantly reduced in both participants. After intervention, both of the participants were able to remain dry for at least 45 min while being engaged in daily activities. Their improvement in saliva control was maintained during follow-up in the home and school environment. Parents’ and teachers’ reports on the VAS and MPS-_saliva control_ gave supportive evidence for the treatment effect.

Anecdotal information from A’s parents and teacher put forward that after the intervention drooling did only sporadically occur with a maximum of once a week. For B, there was a sizable, although not clinically significant, decrease in VAS scores at post-intervention compared to baseline. Also, latency scores for B at the post-intervention and follow-up measurements showed some relapse compared to the end of the centre-based intervention. However, the latency scores remained well above baseline level. Anecdotal reports of her parents and teacher revealed that a cold, which prevented her from breathing through her nose, negatively influenced the saliva control at the time of the 6 weeks follow-up assessment. Since B’s parents and teacher were taught the self-management procedure during initial outpatient intervention, they had the ability to reintroduce self-management training for B after this relapse. Consequently, during the follow-up assessment 24 weeks after treatment, parents reported a renewed decrease in drooling severity on the VAS and also significant increases in the performance and satisfaction regarding saliva control on the MPS-_saliva control_. In addition, anecdotal reports at the follow-up assessment 24 weeks after treatment indicated that the results were also relevant for the teacher.

Besides a cold, there are several alternative physical causes for an increase in drooling severity such as other temporary illnesses, dentition, reflux, growth, etc. Relapse after intervention might also be caused by the participant’s inability to stick to the self-management routine due to distraction by daily issues at school and at home and limited energy to remain focused on saliva control. Alternatively, the participant’s caregiver’s lack of motivation, time or opportunities to continue practice at home may result in relapse (Van der Burg et al. 2016).

Although the outpatient treatment programme yielded promising results, some limitations of the present study are worth noting. As the potential of the outpatient treatment programme was explored in a pair of case studies, there are possible factors (e.g. selection, history, and maturation) that might also have caused the positive increases in saliva control. Consequently, conclusions regarding the effectiveness of the treatment programme are preliminary. Further research with more children suffering from chronic drooling should be carried out before definitive conclusions on the effectiveness of the present treatment programme can be drawn. Both participants in this study were suffering from chronic drooling for almost eight to ten years before inclusion and previous treatments had not provided significant or lasting improvement. For this reason, it is unlikely that the current meaningful increases in their saliva control are the consequence of maturation.

With regard to the data-collection, the maximum time intervals for latency measurements during the study phases might have caused some underestimations of the participants’ ability to remain dry. This is especially relevant for the baseline measurements as an underestimation of the initial ability to remain dry may suggest an unduly positive treatment effect. During baseline A reached maximum latency four times and B reached the maximum latency one time. After intervention, A continued to reach maximum latency scores during post-intervention, and follow-up phases. Therefore, these latency scores may also have been an underestimation of his true ability to remain dry. In addition, visual analysis of the baseline scores of participant A shows a slight upward trend at the end of the baseline sessions. However, given the magnitude of change between the last data points in the baseline phase and the first data points in the intervention phase for both participants, we conclude that the intervention was responsible for the positive change in latency scores.

As the participants were well aware of the purpose of the training, the presence of the trainer during latency measurements might have been a discriminative stimulus to perform self-management behaviour. Clinical anecdotal observations indicated that the presence of the trainer prompted the participants to swallow or check their chins, which might have positively influenced latency scores. Because of these methodological limitations, assessment of social validity of the treatment effect, such as with the VAS and MPS-_saliva control_, are necessary to supplement data on drooling severity from direct observations (i.e., latency scores) in order to reduce the threat to validity.

Another limitation of the present study is that it was not registered how much additional training, feedback, and reinforcement the parents and teachers provided in the home and school situation. Moreover, the number of training sessions provided in the home and school situation did not always correspond to the recommended amount of practice. Parents and teachers of both participants reported that it was sometimes difficult to realise the recommended amount of training sessions because of daily schedules and demands. Consequently, no conclusions can be drawn from this study about the amount of practice, feedback, and reinforcement that was necessary in order to establish and maintain a positive treatment effect.

The time- and cost-intensiveness of an inpatient treatment was the reason to explore the effect of an outpatient behavioural programme for saliva control with continued practice at home and at school. The present study shows that such a treatment programme was feasible and that drooling severity for both children significantly decreased after implementation of the treatment procedure. These results are promising, as they suggest that in addition to centre-based treatment, parent- and teacher-delivered training might be effective in maintaining and reinstating an initial treatment effect in saliva control. This reduces the time spent with professionals in the training for saliva control which allows for a more efficient self-management treatment of chronic drooling.

The present treatment programme still comprised intensive treatment provided by a behavioural therapist for a period of three weeks. For this reason, this outpatient variant of the self-management procedure was still relatively time- and cost-intensive. Moreover, the children had to travel with their parents to and from the centre on a daily basis. Depending on the distance, this may be burdensome or even impossible for some parents. Consequently, not all individuals in need of treatment might be able to receive it. For individuals who do have transportation to and from the rehabilitation clinic, the stress from these daily travels and interruption of the daily affairs of the families might negatively influence the treatment effect. These are considered to be important drawbacks of centre-based treatment. In order to make the self-management programme for saliva control even more feasible and accessible for families with a child suffering from chronic drooling, it is necessary to further investigate the possibility to shorten the child-directed centre-based treatment time without giving in on treatment effectiveness.

Other researchers have sought to adapt original centre-based treatment programmes to fully parent-delivered treatment programmes, although with different target groups. Kroeger and Sorensen (2010) modified an intensive centre-based treatment to a parent-delivered, intensive training protocol implemented within the home-setting for toilet training individuals with autism spectrum disorder (ASD). Parents were verbally instructed on the treatment procedures and received a written protocol. At the first day of treatment, the trainer was present in the child’s house and modelled training procedures for parents. Subsequently, parents implemented the training while the trainer observed and provided coaching. Thereafter, treatment was administered solely by the parents. The findings of Kroeger and Sorensen indicated that caregivers could be successfully instructed to train a child with ASD to independently use the toilet and to maintain this skill over time.

A concern in parent-delivered treatment is the consistency and persistence in providing treatment (Jones 1993). In the present study, the parents and teachers of both children were willing to provide training sessions at home and at school, but they reported that it was sometimes hard to realise the desired amount of training sessions. To help parents and teachers to become proficient and persistent trainers of saliva control by the child, training and support in the use of the self-management procedures in daily routines with the child from a behavioural therapist seem necessary. Similarly to the procedure of Kroeger and Sorensen (2010), one-to-one parent- and teacher-training, preferably in the home or school situation, may be provided. To reduce the logistic drawbacks of one-to-one training, telehealth provisions, such as video-modelling and video-teleconferencing, are considered to be attractive training delivery and behavioural therapist consultancy methods. Vismara et al. (2012) found in their study that the parents of nine toddler-aged children with ASD were successful in learning a parenting intervention programme through telehealth provision, comprising of a DVD learning module and 12 weekly video-conferencing sessions. As many telehealth provisions are available for free, providing treatment or parent-training through telehealth can be cost-effective (Machalicek et al. 2015). Furthermore, the use of telehealth may help the treatment programme to reach out to a greater number of families over a greater distance (Vismara et al. 2012). In the present programme, to support parents and teachers in the application of the treatment procedures at home and at school, the digital healthcare-platform Quli was offered. However, as there was intensive face-to-face contact when the child visited the rehabilitation centre and the trainer could easily be contacted by email or telephone, Quli appeared to be of limited value for parents and teachers in this study. Possibly, a digital healthcare-platform might be of more value in a treatment programme wherein parents and/or teachers are expected to be the main trainers in the self-management procedures. Future research should explore the possibility of a treatment programme for children who suffer from chronic drooling with minimal child-directed training time with a behavioural therapist and the use of telehealth, in order to develop an efficient and affordable intervention for chronic drooling in the natural environment at the child’s home and school.

## Figures and Tables

**Fig. 2:**
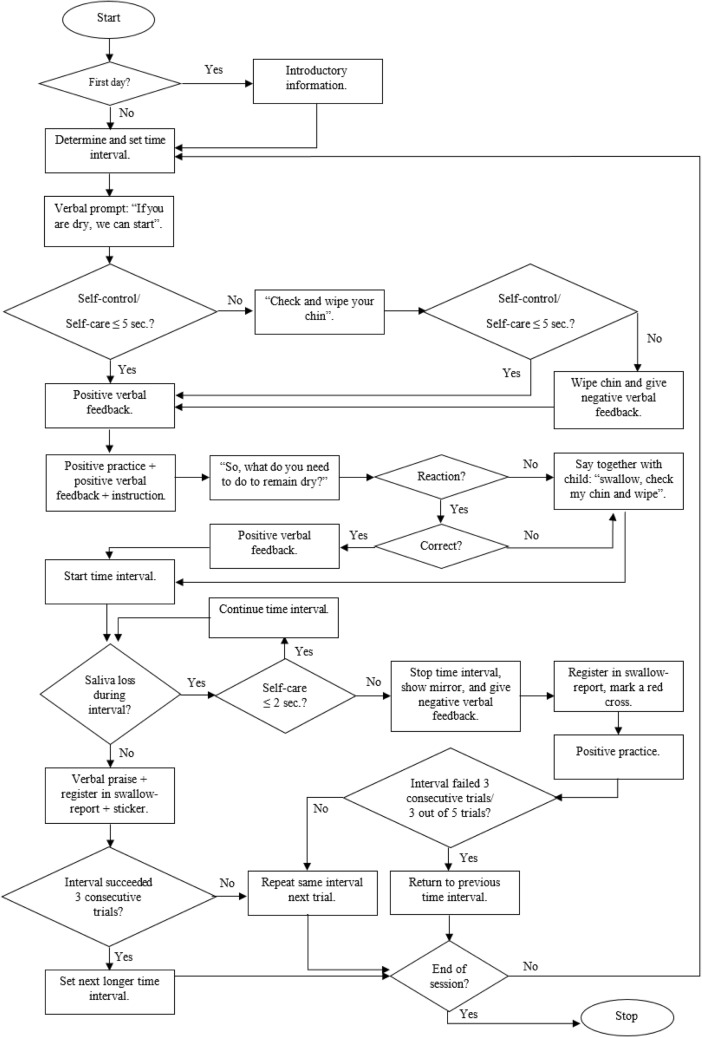
Flowchart of the training procedure for phase 1 training sessions
